# Association between congenital heart defects and maternal manganese and iron concentrations: a case–control study in China

**DOI:** 10.1007/s11356-021-17054-9

**Published:** 2021-12-04

**Authors:** Meixian Wang, Yan Tian, Ping Yu, Nana Li, Ying Deng, Lu Li, Hong Kang, Dapeng Chen, Hui Wang, Zhen Liu, Juan Liang

**Affiliations:** 1 Key Laboratory of Birth Defects and Related Diseases of Women and Children, Ministry of Education, West China Second University Hospital, Sichuan University, Chengdu, China; 2 Liupanshui Maternal and Child Health Care Hospital, Liupanshui Children’s Hospital, Liupanshui, Guizhou China; 3 Chenghua District Maternal and Child Health Hospital of Chengdu, Chengdu, Sichuan China; 4 Mianyang Maternal and Child Health Care Hospital, Mianyang, Sichuan China; 5 National Office for Maternal and Child Health Surveillance of China, West China Second University Hospital, Sichuan University, Chengdu, Sichuan China

**Keywords:** Pregnancy, Congenital heart defect, Manganese, Iron, Maternal exposure, Interaction

## Abstract

To investigate the correlation between maternal manganese and iron concentrations and the risk of CHD among their infant. A multi-center hospital-based case control study was conducted in China. There were 322 cases and 333 controls have been selected from pregnant women who received prenatal examinations. Correlations between CHDs and maternal manganese and iron concentrations were estimated by conditional logistic regression. Moreover, the interaction between manganese and iron on CHDs was analyzed. Compared with the controls, mothers whose hair manganese concentration was 3.01 μg/g or more were more likely to have a child with CHD than those with a lower concentration. The adjusted OR was 2.68 (95%CI = 1.44–4.99). The results suggested that mothers whose iron content was 52.95 μg/g or more had a significantly higher risk of having a child with CHD (*aOR* = 2.87, 95%CI = 1.54–5.37). No interaction between maternal manganese and iron concentrations was observed in the multiplicative or additive model. The concurrently existing high concentration of manganese and iron may bring higher risk of CHD (*OR* = 7.02). Women with excessive manganese concentrations have a significantly increased risk of having offspring with CHDs. The high maternal iron status also correlates with CHDs. The concurrently existing high concentration of manganese and iron may bring higher risk of CHD.

## Introduction

Congenital heart defects (CHDs) are the most common congenital anomalies at birth, accounting for about 28% of major congenital anomalies(Helen et al. 2011; van der Linde et al. 2011). Studies have reported that the incidence of CHD was 1.9/1000 to 9.3/1000 live births(van der Linde et al. 2011; Yingjuan et al. 2019). Although the methods of diagnosis and treatment of CHDs made remarkable progress, CHD is the most common cause of infant mortality in both developing and developed countries (Donghua et al. 2017; Lytzen et al. 2019). The etiology of CHDs still less understood. Genetic abnormalities appear to be the primary cause of CHD; environmental exposures play a role in the pathogenesis. Interactions between genetic variation and environmental exposures results in CHD are complex (Geddes and Earing 2018; Moreau et al. 2019).

Manganese and iron both were essential traced elements. Numerous studies supported that adverse pregnancy outcomes can be compromised by maternal mineral nutrition status’ suboptimization (Lewicka et al. 2019; McAlpine et al. 2019). Manganese plays an important role in fetal development. Neurotoxic effects of high-level exposure to manganese are well established (Lma et al. 2019), but the extent to which environmental exposures may cause adverse health effects to the developing fetus is not well understood (Leonhard et al. 2019; Mora et al. 2015). Animal studies also supported that manganese could accumulate in the heart rapidly to affect the animal’s cardiac muscle cells and cause cardiovascular dysfunction (Crossgrove and Wei 2004; Jiang and Zheng 2005; Yang et al. 2019), exposure to high levels of manganese can result in cardiotoxicity in animals (Shao et al. 2012). Manganese deficiency in the heart may lead to cardiac electrophysiological alterations exhibited as dilated cardiomyopathy (Sunagawa et al. 2014); it is associated with skeleton deformity, reduced reproductive function, and abnormal glucose metabolism, too. Iron overload affected rat heart muscle, resulting in cardiomyopathy (Ward et al. 2009). Studies have found that iron overload leads to heart damages (Rodriguez et al. 2007). However, very few studies have shown that maternal iron deficiency is a risk factor for congenital heart disease(Yang et al. 2020), and no studies reported the association between excess iron or manganese with CHDs. Literatures proved that manganese and iron had a similar orbital size and the coordination number that they shared a common transport site or transport ligand to interact (Keen Carl et al. 2003). These mechanisms can induce heart injuries in humans.

Because of the complicated generating mechanism of CHDs, few epidemiological studies have explored the correlation between manganese and iron concentrations and CHDs. Hair content, as a biomarker of manganese and iron, has been confirmed to be repeatable and reflects a long-term exposure of some substances(Bass et al. 2001; Klevay et al. 2004) that it can be a proper biomarker. ICP-MS is the state of the art in metal analysis to detect multiple metals simultaneously, quickly, and sensitively that it can be used in this study (Elenge et al. 2011).

We conducted a mid-center hospital–based case–control project based on the National Birth Defects Surveillance System in China. Using a detailed questionnaire-based interview to collect the data of pregnant women’s environmental exposures during the periconceptional period, and biological samples were also collected. This article aims to explore that pregnant women’s manganese deficiency or excess raises the risk of having a child with a CHD, and iron excess raises the risk of having a child with a CHD, and to measure the interaction of manganese and iron on the development of the heart preliminarily.

## Method

### Study population

A multi-center hospital-based case–control study was conducted between February 2010 and October 2011 in four tertiary hospitals with the qualification of performing prenatal diagnoses, located in Shenzhen, Zhengzhou, Fuzhou, and Wuhan city, respectively. Participants were recruited from the pregnant women who received prenatal examinations in these four collaborated hospitals. The case group were involved pregnant women whose fetuses were diagnosed with CHDs by echocardiography. In addition, the control group was involved pregnant women whose fetuses were diagnosed without any abnormalities. Once a pregnant woman was included in case group, the first one or two voluntary pregnant women who came from the same hospital during the same study period and whose gestational age differed no more than two weeks with the case was chosen as the proper control. The fetuses of case group were including live births, stillbirths, or abortions, and fetuses of control group were live births.

Exclusion criteria for our project were as follows: (1) multiple pregnancy, (2) gestational age over than 14 weeks, (3) fetuses of case group who had the CHD associated with chromosome disorders or single-gene disorders, (4) fetuses of control group who had any abnormalities, and (5) fetuses with unclear diagnosis. To comply with the purpose of this article, several additional exclusion criteria were set as follows: (1) pregnant women who have color-treated or over-processed hair; (2) pregnant women with mental disorders; (3) fetuses of case group had the CHD associated with any extracardiac malformation.

### Determination and classification of CHDs

The diagnosis of each live birth child was confirmed within three months after birth by specialists in the areas of ultrasound, obstetrics, and pediatrics in each hospital, and that of stillbirths and terminations were confirmed through autopsy reports. Furthermore, to ensure the accuracy of the diagnosis, all static and dynamic echocardiography images of CHDs cases were reviewed by 4–5 state-level prenatal ultrasound specialists and pediatric cardiologists.

Each child of the case group was had at least one kind of cardiac lesion without any other non-CHD anomalies. In line with anatomic lesion and clinical classification, the cases of CHDs in our study were divided into six major categories. For those cases of CHDs which had more than one category, they were double-counted in this study, included the following: (1) septal defects, including atrial septal defects(ASDs) and ventricular septal defect(VSDs), atrioventricular septal defects (AVSDs), and endocardial cushion defect(ECD); (2) conotruncal defects, including transposition of the great arteries, tetralogy of Fallot, truncus arteries, double-outlet right ventricle; (3) left ventricular outflow tract obstruction, including aortic valve stenosis, hypoplastic left heart syndrome and variants, coarctation of the aorta, and interrupted aortic arch types A and C; (4) right ventricular outflow tract obstruction, including pulmonary valve stenosis, pulmonary atresia, tricuspid atresia, and Ebstein anomaly; (5) anomalous pulmonary venous return, including total and partial anomalous pulmonary venous return; (6) other heart defects (Malik et al. 2008).

### Hair sample collection and laboratory assessment

Mothers’ hair was collected when they were initially included in the study, and the gestational week is between 24 and 28 weeks. The hair strand was cut at scalp level, which was about 3–5 cm long, weighed about 1 g and as coarse as a pencil from the occipital area of the pregnant woman using stainless steel scissors by trained specimen collectors. Each 100 mg of the hair sample was microwave-digested (CEM) at 180 °C following the manufacturing procedure, and microwave-digested tubes were added 5 mL HNO_3_. Then, these tubes were heated to near dryness on a heating plate, and subsequently diluted with 2% HNO_3_ up to 2 ml. The diluted samples were stored at 4 °C for ICP-MS analysis.

We used the Agilent 7500 CX ICP/MS system (Agilent Technologies, Wilmington, DE) equipped with a G3160B I-AS integrated Autosampler to perform the ICP-MS. The instrument settings and procedures were the same as used in an earlier study (Sun et al. 2012). The internal standard was the multi-element standard solutions (SPEX CertiPrep, Metuchen, NJ), and it was injected by the peristaltic pump into the ion source at an approximate concentration of 500 μg/L in the online mode. Ultrapure water (18.2 MΩ) was obtained from a water-purification system (Sartorius, arum 61,316). Ultrapure grade HNO_3_ (100 port, 65% v/v, TAMA) was used in this study.

This study was approved by the Medical Ethics Committee of Sichuan University (No.2010004). All mothers signed the informed consents.

### Questionnaire

According to literature reviews and expert advices, we designed a detailed and structured questionnaire to collect information about pregnant women and their husbands’ demographic characteristics and multiple environmental exposures during the pregnancy period. Every person who was willing to participate in the study signed an informed consent form and received a face-to-face interview to complete each questionnaire by a trained investigator.

### Statistical analysis

Maternal characteristics were described by median (interquartile range) (skewness distribution data) or numbers (%). The composition ratio of maternal characteristics between case and control groups was compared by the Mann–Whitney *U* test and *χ*^2^ test. The distributions of manganese and iron concentrations were presented at the median (inter-quartile range, IQR) because of their non-normal distributions (were still non-normal after log-transformed). Comparisons among case and control groups were calculated by the Mann–Whitney *U* test.

To measure the correlation between concentrations of manganese and iron and fetal CHDs, the multivariate logistic regressions were used to calculate the adjusted odds ratios (*aOR*) and their 95% confidence intervals (95% CI). Factors with statistically significant will be taken into adjusted model analysis.

The normal range of manganese and iron concentrations in maternal hair was still unknown, so the 5th to 95th percentile of the control group’s (*n* = 333) manganese and iron concentrations were used as reference values in the logistic regressions. The other two tails represented the lower or higher concentration section, respectively. Potential confounding factors were chosen according to the researches and ascertained using statistical methods, including maternal age (age at the last menstrual period), maternal residence (urban, suburbs or rural), gestational age (age at the beginning of study enrollment), the number of weeks of folic acid taken after pregnancy in this study. Those three factors were brought into the logistic regressions by the form of continuous variation. Exposure to a factory, landfill nearby home or workplace (< 1000 m) was shown as a potential source of exposure (outside exposure).

The additive interaction was calculated by a four-by-two table and an Excel (Andersson et al. 2005; Zou 2008), and the multiplicative interaction was calculated by the binary logistic regression. Before testing the hypothesis of interactions, we defined the subjects’ exposed and unexposed status: the low and medium sections of manganese or iron were merged as an unexposed group since no significant correlations were observed with low concentrations, and the high section of manganese or iron was defined as the exposed group. The RERI (relative excess risk due to interaction) and its 95%CI have been used as the measures of additive interaction; if there was no additive interaction between manganese and iron, the confidence intervals of RERI include 0 (Hu et al. 2015).

Data were statistically analyzed using SPSS v.20.0 (SPSS Inc., Chicago: IL) and Microsoft Excel 2010 (Microsoft Corporation, Redmond, Washington). The significance level was 0.05.

## Results

In this project, 152 fetuses of case group who had chromosome disorders, single-gene disorders, other extracardiac malformations or unclear diagnoses have been excluded. Fifty mothers of control group have been excluded due to lost follow-up. Moreover, 59 hair samples of cases group and 34 of control group were color-treated or over-processed. Furthermore, there were 60 mothers’ manganese or iron concentrations of were lower than the detection limit (33 of case group and 27 of control group, respectively). Finally, we got 322 cases and 333 controls in this study.

In case group, septal defects were the most frequent malformations (145, 45.03%) followed by conotruncal defects (144, 44.72%), right ventricular outflow tract obstruction (74, 22.98%), left ventricular outflow tract obstruction (61, 18.94%), anomalous pulmonary venous return (54, 16.77%), and other heart defects (50, 15.53%).

Table 1 shows the maternal characteristics of participants from both groups. The maternal BMI, the first pregnancy, and city were similar in both groups, but the results showed significant differences in maternal age, gestational age, the number of weeks of folic acid taken after pregnancy, maternal residence, and outside exposure. The 5 factors will been taken into adjusted in multivariate analysis.

**Table 1:** The maternal characteristics of case and control participants

Variable	Cases (*n* = 322)	Controls (*n* = 333)	*Z /* χ^2^	*P* value
Maternal age				
Median (interquartile range)	27.00(6.00)	28.00(6.00)	-2.13	0.033^*^
Maternal BMI				
Median (interquartile range)	23.44(3.89)	23.14 (4.41)	0.39	0.679
The number of weeks of folic acid taken after pregnancy				
Median (interquartile range)	12.00(12.00)	12.00(8.00)	-3.13	0.002^*^
Gestational age				
Median (interquartile range)	26.00(6.93)	24.86(4.86)	2.46	0.014^*^
Maternal residence				
Urban	190 (59.01)	263(78.98)	37.99	< 0.001^*^
Suburbs	88 (27.33)	59 (17.72)		
Rural	41 (12.73)	9 (2.70)		
Outside exposure				
Yes	96 (29.81)	51 (15.32)	19.77	< 0.001^*^
No	266 (70.19)	282(84.68)		
First pregnancy				
Yes	129(40.06)	160 (48.05)	5.13	0.077
No	192 (59.63)	173 (51.95)		
City				
Fujian	39(38.61)	62(61.39)	5.64	0.131
Hubei	58 (53.21)	51 (46.79)		
Henan	64 (51.61)	60 (48.39)		
Shenzhen	161 (50.16)	160(49.84)		

Data are median (interquartile range) or numbers (%)

^a^Obtained using the Mann–Whitney *U* test for maternal age, maternal BMI, the number of weeks of folic acid taken after pregnancy and gestational age; the *χ*^2^ test for maternal residence, outside exposure, first pregnancy, and city

^*^Significant results, *P* value < 0.05

We compared the median and IQR levels of hair manganese and iron concentrations between the case and control groups, and case subgroups and control group (Table 2). The case and control groups’ manganese median (interquartile range, IQR) concentrations were 1.04 (0.54–2.170) μg/g and 0.43 (0.24–0.80) μg/g, respectively, and the case and control groups’ iron median (interquartile range, IQR) concentrations were 22.42 (13.90–36.55) μg/g and 13.56 (8.87–20.07) μg/g, respectively. Manganese’s reference range was 0.11–3. 01 μg/g, and iron’s was and 3.15–52.95 μg/g. There were significant differences in the concentration distribution of manganese and iron between the case group and the control group, and the case subgroup and the control group.

**Table 2:** The concentrations (median, *P*_5_, *P*_25_, *P*_75_, and *P*_95_) of manganese and iron in case and control groups

	Manganese(μg/g)							Iron(μg/g)						
	Median	*P*_5_	*P*_25_	*P*_75_	*P*_95_	*Z*	*P* value	Median	*P*_5_	*P*_25_	*P*_75_	*P*_95_	*Z*	*P* value
Controls	0.43	0.11	0.24	0.80	3.01	Ref	Ref	13.56	3.15	8.87	20.07	52.95	Ref	Ref
Cases	1.04	0.18	0.54	2.17	5.78	− 9.74	< 0.001^*^	22.42	4.45	13.90	36.55	125.60	− 8.40	< 0.001^*^
Septal defects^b^	1.00	0.21	0.54	2.17	7.00	− 7.50	< 0.001^*^	21.16	2.94	12.56	35.59	122.69	− 5.65	< 0.001^*^
Conotruncal defects^b^	1.08	0.20	0.54	2.26	6.18	− 7.98	< 0.001^*^	21.80	3.06	13.80	34.29	130.16	− 6.27	< 0.001^*^
Left ventricular outflow track obstruction^b^	1.11	0.10	0.54	2.16	9.45	− 5.64	< 0.001^*^	23.07	4.70	13.74	44.05	151.03	− 5.28	< 0.001^*^
Right ventricular outflow track obstruction^b^	1.00	0.11	0.58	2.02	5.53	− 5.11	< 0.001^*^	22.28	5.66	15.56	31.92	60.82	− 5.19	< 0.001^*^

^a^Mann-Whitney *U* test was used for the comparison between the case and control groups and the comparisons between the CHD subgroups and control group

^b^The cases of CHDs in our study were divided into six major subgroups, and the first four groups’ concentrations had been shown

^*^Significant results, *P* value < 0.05

Table 3 shows the correlation between maternal manganese and iron concentrations and CHDs, for all CHDs and each group of heart defect subtypes. Mothers characterized with high manganese concentration were more likely to have children with CHDs, compared with mothers who had the medium manganese concentration (*aOR* = 2.68, 95%CI = 1.44–4.99). This correlation was observed in mothers with the high iron concentration (*aOR* = 2.87, 95%CI = 1.54–5.37). High iron concentration was a great risk factor for having children with the left ventricular outflow tract obstruction, compared with mothers who had the medium iron concentration (*aOR* = 4.81, 95%CI = 2.18–10.61). Due to smaller sample size, the result of subgroup of CHD should be treated with caution.

**Table 3:** Multivariate analysis of maternal manganese and iron concentrations and CHD occurrence

CHD subtype	Manganese(μg/g)									iron(μg/g)								
	Low (≤ 0.11)				High (≥ 3.01)				Medium^a^ (0.11–3.01)	Low (≤ 3.15)				High (≥ 52.95)				Medium^a^ (43.15–52.95)
	*N*	*β*	*P* value	*aOR* ^b^ (95% CI)	*N*	*β*	*P* value	*aOR*^b^ (95% CI)	*N*	*N*	*β*	*P* value	*aOR*^c^ (95% CI)	*N*	*β*	*P* value	*aOR*^c^ (95% CI)	*N*
Controls	15	Ref	Ref	Ref	16	Ref	Ref	Ref	302	16	Ref	Ref	Ref	16	Ref	Ref	Ref	301
All CHDs	10	− 0.11	0.798	0.90 (0.39–2.08)	47	0.98	0.002*	2.68 (1.44–4.99)	265	10	− 0.40	0.372	0.67 (0.28–1.61)	41	1.06	0.001*	2.87 (1.54–5.37)	271
Septal defects	3	− 0.54	0.405	0.58 (0.16–2.09)	19	0.85	0.024*	2.33 (1.12–4.86)	123	7	0.06	0.903	1.06 (0.40–2.86)	19	1.08	0.003*	2.95 (1.43–6.09)	119
Conotruncal defects	2	—^d^	—^d^	—^d^	22	1.04	0.004*	2.84 (1.40–5.76)	120	7	0.02	0.972	1.02 (0.39–2.69)	14	0.79	0.043*	2.20 (1.02–4.74)	123
Left ventricular outflow tract obstruction	5	0.82	0.138	2.27 (0.77–6.70)	12	1.16	0.007*	3.19 (1.38–7.38)	57	2	—^d^	—^d^	—^d^	15	1.57	0.000*	4.81 (2.18–10.61)	57
Right ventricular outflow tract obstruction	3	0.29	0.665	1.33 (0.36–4.94)	9	1.07	0.020*	2.91 (1.19–7.14)	49	0	—^d^	—^d^	—^d^	4	0.25	0.676	1.28 (0.40–4.06)	57

^a^Medium is the reference group

^b^Adjusted for mother’s age, gestational age, the number of weeks of folic acid taken after pregnancy, maternal residence, and outside exposure for manganese

^c^Adjusted for mother’s age, gestational age, the number of weeks of folic acid taken after pregnancy, maternal residence, and outside exposure for iron

^d^Multivariable model results in which less than 3 cases exposed are not shown

^*^Significant results, *P* value < 0.05

After adjustment for potential confounding variables, there was no significant multiplicative interaction between the combined factors (manganese and iron) and CHDs (*P* value = 0.492 > 0.05). The RERI was 4.015 (95%CI =  − 5.012 to 13.042), indicating that there was no additive interactions between manganese and iron for CHDs. We found that the concurrently existing high concentration of manganese and iron may bring higher risk of CHD (*OR* = 7.02) (see Table 4).

**Table 4:** The interaction analysis between manganese and iron concentrations for CHD

Manganese	Iron	Case(n)	Control(*n*)	*β*	*P* value	*aOR* (95% CI)^a^
Medium + low	Medium + low	251	304	—	—	Ref
High	Medium + low	24	13	0.75	0.044^*^	2.11(1.02–4.37)
Medium + low	High	30	13	0.64	0.085	1.89(0.92–3.90)
High	High	17	3	1.95	0.003^*^	7.02(1.98–24.85)

^a^Adjusted for mother’s age, gestational age, the number of weeks of folic acid taken after pregnancy, maternal residence, and outside exposure for manganese

^*^Significant results, *P* value < 0.05

### Discussion

In our study, a significant correlation has been observed between maternal high manganese concentration and CHDs. Similar results have been observed between maternal high iron concentration and CHDs, and our results revealed that the concurrently existing high concentration of manganese and iron may bring higher risk of CHD.

Manganese and iron are essential for proper fetal development. An active maternal–fetal transport mechanism for manganese is dominant, which means pregnant women’s manganese status may thus represent that of their fetus (Miyoung et al. 2009; Nandakumaran et al. 2016). The routes of manganese exposure for pregnant women may be environmental exposure to manganese in atmospheric contamination, dietetic exposure in water or food and occupational exposure to airborne manganese in welding or smelter (Hoet et al. 2012; Tupwongse et al. 2007). Manganese overexposure has been proved to have neurotoxicity and cardiovascular toxicities to adults(Crossgrove and Wei 2004; Magari et al. 2002), but little is known about the relationship between manganese overload and pregnancy outcomes, especially CHDs. Early radiotracer studies suggest that iron is transported actively from the mother to the fetus and fetal iron storage may be more than maternal storage. Once iron is transferred to the fetus, there is no recycling of nonheme iron from fetal to maternal circulation (Cao and O'Brien 2013; Gambling et al. 2011; Mcardle and Morgan 1982; Wong and Morgan 1973), so maternal iron status represents fetal iron status approximately. The excessive iron of pregnant women may come from drinking water or food, environmental contamination and occupation (Connell et al. 2006; Tupwongse et al. 2007). Some genetic disorders such as hereditary hemochromatosis also lead to iron overload (Rodriguez et al. 2007). It is reasonable to measure the maternal manganese and iron concentrations as a biomarker to explore the correlation with CHD.

Animal studies have proven that manganese can accumulate in the myocardial mitochondria and inhibit mitochondrial energy production through damaging its structure and function, such as causing mitochondrial vacillation, restraining the function of the proton pump, and reducing the mitochondrial membrane potential (Jiang and Zheng 2005). These altered functions contributed to the decline of intracellular ATP concentrations, apoptosis of myocardial cells, a decline in cardiac contraction, and a change in heart rate(Gunter et al. 2010; Jiang and Zheng 2005; Yang et al. 2010). Another study also found that heart tissue had infiltration of inflammatory cells, disordered or frosted myocardium fibers, conduct hypertrophy and vascular degeneration after manganese treatment (Shao et al. 2012). Epidemiologic studies suggest an association between manganese overloaded and significant alterations in cardiac autonomic nerve function to indirectly affect cardiovascular function such as a decrease in parasympathetic high frequency activation of heart rate variability(He et al. 2004; Magari et al. 2002). Presumably, these underlying mechanisms relate to the induction of CHDs, by excessive manganese. Our study showed mothers whose hair manganese concentration was high (3.01 μg/g or more) were more likely to have a child with CHD than those with a lower concentration.

Heart asylums and aldehyde concentrations (lipid peroxidation products, markers of oxidative stress) were observed significant dose-dependent increases and positive correlations with iron administered in a chronic iron exposed murine model, oxidative stress may cause cardiac failure in mice (Davis and Bartfay 2004). Animal studies have also found that in the heart tissue, iron overload would raise the expression of glutathione peroxidase 3, a protective mechanism against oxidative stress-induced damage. The expression of genes encoding calcium- and zinc-binding proteins, S100a8 (calgranulin A) and S100a9 (calgranulin B), proteins play a role in oxidative stress response; the expression of pyruvate dehydrogenase kinase 4 (Pdk 4), an enzyme that may cause diabetes mellitus to result in cell damage; the expression of angiopoietin-like 4 (Angptl4), a secreted protein can lead to reduced cardiac LPL activity in heart muscle and cardiomyopathy (Carlsson et al. 2005; Rodriguez et al. 2007). During oxidative stress, damage in the heart can be induced to cell constituents such as mitochondrial membrane constituents in rat hearts; this type of damage will lead to mitochondrial respiration dysfunction and lead to heart reperfusion injuries (Khaliulin et al. 2004). Iron overload disorders may also activate the interstitial fibroblast to induce cardiac fibrosis (Cheung et al. 2015; Das et al. 2017). These phenomena may occur not only in adults but also in fetuses to induce CHDs. We detected mothers whose iron content high (52.95 μg/g or more) had a significantly higher risk of having a child with CHD.

We measured the interaction of manganese and iron on CHD in our study. Manganese and iron share a common absorption pathway in the gut; the influence of Mn on Fe homeostasis may be mediated through its influence on Fe absorption, circulating transporters like transferrin, and regulatory proteins (Altstatt et al. 1967; Bjørklund et al. 2017). Chronic manganese exposure expedited unidirectional influx of iron from the systemic circulation to the cerebral compartment which means manganese may interact with iron at cellular iron uptake. This mechanism makes manganese induce more impairment in cellular regulatory processes and generates oxidative stress, and makes iron generate lipid peroxidation and iron-mediated reactive oxygen species (Wei et al. 1999). Animal studies have found that no matter iron depletion or overload, increases manganese concentrations in the brain, this synergistic interaction occurs during the transfer them from the plasma to other organs to damage tissue (Chua and Morgan 1996). Similar results (parenteral administration of iron resulted in a significant increase of manganese in the liver and spleen) have been observed in chicks (Baker and Halpin 1991). No interaction between maternal manganese and iron concentrations was observed in our study. However, we found that the concurrently existing high concentration of manganese and iron may bring significant higher risk of CHD (*OR* = 7.02). An interaction between manganese and iron might exist, but an epidemiological study with a larger sample size is needed to confirm.

### Strength and limitation

The pregnant women’s manganese and iron concentrations were measured by using hair as the biomarker since it is repeatable, collecting and storing easily, and can reflect a long-term exposure of metals. The method used to detect manganese and iron concentrations, and the classification of CHDs was consistent with other researches on a global scale. Some CHDs are fatal (such as tetralogy of Fallot and pulmonary atresia with VSD), and live births, stillbirths, and terminations with more kinds of CHDs have been involved in our case fetuses. Therefore, the CHD disease spectrum of our subjects is more similar and relevant to the human CHD disease spectrum than other studies.

There are also limitations in this study. First, due to the different ethnic groups, residences, and determination methods in previous studies, the reference values of hair metal concentrations were various, and the normal ranges of manganese and iron concentrations in pregnant women were unknown neither. Therefore, the 5th to the 95th percentile of manganese and iron concentrations in the control group were used as reference values. Second, as a hospital-based case–control study, admission rate bias was inevitable, so our subjects selected were different from the CHDs randomly chosen from the general population.

Consideration of potential factors is another limitation in this study. Because some data information is restricted to use, the controlled confounders taken into multiple logistics regression were not enough, and our sample size is relatively small; analysis on subgroup of CHD should be treated with caution; further studies are needed to prove our results.

We collected maternal hair to test iron level in hair, but did not association between CHD and source of sample including iron or manganese, such as maternal blood sample. More research will be done in the future on the correlation between CHD and other different biological samples including iron or manganese.

## Conclusions

In conclusion, high maternal manganese concentration correlates with the occurrence of CHDs in offspring. High maternal iron concentration is also suggested to correlate with the occurrence of CHDs in offspring. Although the interaction between manganese and iron for CHDs was not observed in this study, the concurrently existing high concentration of manganese and iron may bring higher risk of CHD; further epidemiological studies using a larger sample size are needed to perform.

## Data Availability

All data generated or analyzed during this study are included in this published article.

## References

[CR1] Altstatt LB, Pollack S, Feldman MH, Reba RC, Crosby WH (1967) Liver manganese in hemochromatosis. Proc Soc Exp Biol Med 124:353–355. 10.3181/00379727-124-31741 10.3181/00379727-124-31741 6019870

[CR2] Andersson T, Alfredsson L, Kallberg H, Zdravkovic S, Ahlbom A (2005) Calculating measures of biological interaction. Eur J Epidemiol 20:575–579. 10.1007/s10654-005-7835-x 10.1007/s10654-005-7835-x 16119429

[CR3] Baker DH, Halpin KM (1991) Manganese and iron interrelationship in the chick. Poult Sci 70:146–52. 10.3382/ps.0700146 10.3382/ps.0700146 2017410

[CR4] Bass DA, Hickock D, Quig D, Urek K (2001) Trace element analysis in hair: Factors determining accuracy, precision, and reliability. Altern Med Rev 6:472–481 11703167

[CR5] Bjørklund G, Aaseth J, Skalny AV, Suliburska J, Skalnaya MG, Nikonorov AA, Tinkov AA (2017). Interactions of iron with manganese, zinc, chromium, and selenium as related to prophylaxis and treat ment of iron deficiency. J Trace Elem Med Biol.

[CR6] Cao C, O'Brien KO (2013) Pregnancy and iron homeostasis: an update. Nutr Rev 71:35–51. 10.1111/j.1753-4887.2012.00550.x 10.1111/j.1753-4887.2012.00550.x 23282250

[CR7] Carlsson H, Yhr M, Petersson S, Collins N, Polyak K, Enerbäck C (2005) Psoriasin (S100A7) and calgranulin-B (S100A9) induction is dependent on reactive oxygen species and is downregulated by Bcl-2 and antioxidants. Cancer Biol Ther 4:998–1005. 10.4161/cbt.4.9.1969 10.4161/cbt.4.9.1969 16082188

[CR8] Cheung YF, Lam WW, Ip JJ, Cheuk DK, Cheng FW, Yang JY, Yau JP, Ho KK, Li CK, Li RC, Yuen HL, Ling AS, Li VW, Chan GC (2015). Myocardial iron load and fibrosis in long term survivors of childhood leukemia. Pediatr Blood Cancer.

[CR9] Chua AC, Morgan EH (1996) Effects of iron deficiency and iron overload on manganese uptake and deposition in the brain and other organs of the rat. Biol Trace Elem Res 55:39–54. 10.1007/BF02784167 10.1007/BF02784167 8971353

[CR10] Connell DP, Winter SE, Conrad VB, Kim M, Crist KC (2006) The Steubenville Comprehensive Air Monitoring Program (SCAMP): concentrations and solubilities of PM(2.5) trace elements and their implications for source apportionment and health research. J Air Waste Manag Assoc 56:1750–1766. 10.1080/10473289.2006.10464580 10.1080/10473289.2006.10464580 17195494

[CR11] Crossgrove J, Wei Z (2004) Manganese toxicity upon overexposure. NMR Biomed 17:544–553. 10.1002/nbm.931 10.1002/nbm.931 PMC3980863 15617053

[CR12] Das SK, Patel VB, Basu R, Wang W, DesAulniers J, Kassiri Z, Oudit GY (2017) Females are protected from iron-overload cardiomyopathy independent of iron metabolism: key role of oxidative stress. J Am Heart Assoc 6. 10.1161/JAHA.116.003456 10.1161/JAHA.116.003456 PMC5523622 28115312

[CR13] Davis MT, Bartfay WJ (2004) Dose-dependent effects of chronic iron burden on heart aldehyde and acyloin production in mice. Biol Trace Elem Res 99:255–268. 10.1385/BTER:99:1-3:255 10.1385/BTER:99:1-3:255 15235157

[CR14] Elenge MM, Aubry JC, Jacob L, De Brouwer C (2011) Heavy metal in hair samples of 109 non-industrial (miners) population in Katanga. Sante 21:41–46. 10.1684/san.2011.0229 10.1684/san.2011.0229 21700541

[CR15] Gambling L, Lang C, McArdle HJ (2011). Fetal regulation of iron transport during pregnancy. Am J Clin Nutr.

[CR16] Geddes GC, Earing MG (2018) Genetic evaluation of patients with congenital heart disease. Pediatr 30:707–713. 10.1097/MOP.0000000000000682 10.1097/MOP.0000000000000682 PMC6257101 30138133

[CR17] Gunter TE, Gerstner B, Lester T, Wojtovich AP, Malecki J, Swarts SG, Brookes PS, Gavin C, Gunter KK (2010) An analysis of the effects of Mn2+ on oxidative phosphorylation in liver, brain, and heart mitochondria using state 3 oxidation rate assays. 249:65–75. 10.1016/j.taap.2010.08.018 10.1016/j.taap.2010.08.018 PMC3004221 20800605

[CR18] He SC, Niu Q (2004) Subclinical neurophysiological effects of manganese in welding workers. Int J Immunopathol Pharmacol 17:11–6. 10.1177/03946320040170S203 10.1177/03946320040170S203 15345186

[CR19] Helen D, Maria L, Group ESoCAEW (2011) Congenital Heart Defects in Europe: Prevalence and Perinatal Mortality, 2000 to 2005. Circulation 123 10.1161/CIRCULATIONAHA.110.958405 21321151

[CR20] Hoet P, Vanmarcke E, Geens T, Deumer G, Haufroid V, Roels HA (2012) Manganese in plasma: a promising biomarker of exposure to Mn in welders. A pilot study. Toxicol Lett 213:69–74. 10.1016/j.toxlet.2011.06.013 10.1016/j.toxlet.2011.06.013 21704687

[CR21] Hu H, Liu Z, Li J, Li S, Zhu J (2015) Correlation between congenital heart defects and maternal copper and zinc concentrations. Birth Defects Res A Clin Mol Teratol 100. 10.1002/bdra.23284 10.1002/bdra.23284 25131520

[CR22] Jiang Y, Zheng W (2005) Cardiovascular toxicities upon manganese exposure. Cardiovasc Toxicol 5:345. 10.1385/ct:5:4:345 10.1385/ct:5:4:345 PMC3980854 16382172

[CR23] Keen CL, Clegg MS, Hanna LA, Lanoue L, Rogers JM, Daston GP, Oteiza P, Uriu-Adams JY (2003) The plausibility of micronutrient deficiencies being a significant contributing factor to the occurrence of pregnancy complications. J Nutr 133 10.1093/jn/133.5.1597S 12730474

[CR24] Khaliulin I, Schneider A, Houminer E, Borman JB, Schwalb H (2004) Apomorphine prevents myocardial ischemia/reperfusion-induced oxidative stress in the rat heart. 37:969–976. 10.1016/j.freeradbiomed.2004.06.029 10.1016/j.freeradbiomed.2004.06.029 15336313

[CR25] Klevay LM, Christopherson DM, Shuler TR (2004) Hair as a biopsy material: trace element data on one man over two decades. Eur J Clin Nutr 58:1359–1364. 10.1038/sj.ejcn.1601975 10.1038/sj.ejcn.1601975 15069459

[CR26] Leonhard MJ, Chang ET, Loccisano AE, Garry MR (2019). A systematic literature review of epidemiologic studies of developmental manganese exposure and neuro developmental outcomes. Toxicology.

[CR27] Lewicka I, Kocyłowski R, Grzesiak M, Gaj Z, Sajnóg A, Barałkiewicz D, von Kaisenberg C, Suliburska J (2019). Relationship between pre-pregnancy body mass index and mineral concentrations in serum and amniotic fluid in pregnant women during labor. J Trace Elem Med Biol.

[CR28] Liu Y, Chen S, Zühlke L, Black G, Mun-Kit (2019) Global birth prevalence of congenital heart defects 1970-2017: updated systematic review and meta-analysis of 260 studies. Int J Epidemiol 48:455–463. 10.1093/ije/dyz009 10.1093/ije/dyz009 PMC6469300 30783674

[CR29] Lytzen R, Vejlstrup N, Bjerre J, Petersen OB, Leenskjold S, Dodd JK, Jorgensen FS, Sondergaard L (2019) Mortality and morbidity of major congenital heart disease related to general prenatal screening for malformations. Int J Cardiol 290:93–99. 10.1016/j.ijcard.2019.05.017 10.1016/j.ijcard.2019.05.017 31130278

[CR30] Magari SR, Schwartz J, Williams PL, Hauser R, Smith TJ, Christiani DC (2002) The association of particulate air metal concentrations with heart rate variability. Environ Health Perspect 110:875–880. 10.1289/ehp.02110875 10.1289/ehp.02110875 PMC1240986 12204821

[CR31] Malik S, Cleves MA, Honein MA, Romitti PA, Botto LD, Yang S, Hobbs CA (2008) Maternal smoking and congenital heart defects. Pediatrics 63:e810–6. 10.1542/peds.2007-1519 10.1542/peds.2007-1519 18381510

[CR32] McAlpine JM, McKeating DR, Vincze L, Vanderlelie JJ, Perkins AV (2019). Essential mineral intake during pregnancy and its association with maternal health and birth outcomes in South East Queensland, Australia. Nutr Metab Insights.

[CR33] McArdle HJ, Morgan EH (1982) Transferrin and iron movements in the rat conceptus during gestation. J Reprod Fertil 66:529–536. 10.1530/jrf.0.0660529 10.1530/jrf.0.0660529 7175808

[CR34] Mezzaroba L, Alfieri DF, Colado Simão AN, EM VR (2019): The role of zinc, copper, manganese and iron in neurodegenerative diseases. Neurotoxicology 74:230–241. 10.1016/j.neuro.2019.07.007 10.1016/j.neuro.2019.07.007 31377220

[CR35] Miyoung Y, Nong A, Clewell HJ, Taylor M, Dorman DC, Andersen ME (2009) Evaluating placental transfer and tissue concentrations of manganese in the pregnant rat and fetuses after inhalation exposures with a PBPK model. Toxicol Sci 112:44–58. 10.1093/toxsci/kfp198 10.1093/toxsci/kfp198 19726578

[CR36] Mora AM, van Wendel de Joode B, Mergler D, Córdoba L, Cano C, Quesada R, Smith DR, Menezes-Filho JA, Eskenazi B (2015). Maternal blood and hair manganese concentrations, fetal growth, and length of gestation in the ISA co hort in Costa Rica. Environ Res.

[CR37] Moreau JLM, Kesteven S, Martin E, Lau KS, Yam MX, O'Reilly VC, Del Monte-Nieto G, Baldini A, Feneley MP, Moon AM, Harvey RP, Sparrow DB, Chapman G, Dunwoodie SL (2019) Gene-environment interaction impacts on heart development and embryo survival. Development 146. 10.1242/dev.172957 10.1242/dev.172957 30787001

[CR38] Nandakumaran M, Al-Sannan B, Al-Sarraf H, Al-Shammari M (2016). Maternal-fetal transport kinetics of manganese in perfused human placental lobule in vitro. J Matern Fetal Neonatal Med.

[CR39] Rodriguez A, Hilvo M, Kytömäki L, Fleming RE, Britton RS, Bacon BR, Parkkila S (2007) Effects of iron loading on muscle: genome-wide mRNA expression profiling in the mouse. BMC Genomics 8:379. 10.1186/1471-2164-8-379 10.1186/1471-2164-8-379 PMC2151772 17949489

[CR40] Shao JJ, Yao HD, Zhang ZW, Li S, Xu SW (2012). The disruption of mitochondrial metabolism and ion homeostasis in chicken hearts exposed to manganese. Toxicol Lett.

[CR41] Sun L, Yu Y, Huang T, An P, Yu D, Yu Z, Li H, Sheng H, Cai L, Xue J (2012) Associations between ionomic profile and metabolic abnormalities in human population. PLoS One 7:e38845. 10.1371/journal.pone.0038845 10.1371/journal.pone.0038845 PMC3374762 22719963

[CR42] Sunagawa T, Shimizu T, Matsumoto A, Tagashira M, Kanda T, Shirasawa T, Nakaya H (2014) Cardiac electrophysiological alterations in heart/muscle-specific manganese-superoxide dismutase-deficient mice: prevention by a dietary antioxidant polyphenol. Biomed Res Int 2014:704291. 10.1155/2014/704291 10.1155/2014/704291 PMC3977505 24772433

[CR43] Tupwongse V, Parkpian P, Watcharasit P, Satayavivad J (2007) Determination of levels of Mn, As, and other metals in water, sediment, and biota from Phayao Lake, Northern Thailand, and assessment of dietary exposure. Environ Lett 42:1029–1041 10.1080/10934520701418458 17616874

[CR44] van der Linde D, Konings EE, Slager MA, Witsenburg M, Helbing WA, Takkenberg JJ, Roos-Hesselink JW (2011) Birth prevalence of congenital heart disease worldwide: a systematic review and meta-analysis. J Am Coll Cardiol 58:2241-7. 10.1016/j.jacc.2011.08.025 10.1016/j.jacc.2011.08.025 22078432

[CR45] Ward RJ, Wilmet S, Legssyer R, Leroy D, Toussaint L, Crichton RR, Pierreux C, Hue L, Piette J, Srai SK, Solanky N, Klein D, Summer K (2009) Effects of marginal iron overload on iron homeostasis and immune function in alveolar macrophages isolated from pregnant and normal rats. Biometals 22:211–223. 10.1007/s10534-008-9155-6 10.1007/s10534-008-9155-6 18690415

[CR46] Wei Z, Zhao Q, Slavkovich V, Aschner M, Graziano JH (1999) Alteration of iron homeostasis following chronic exposure to manganese in rats. Brain Res 833:125–32 10.1016/s0006-8993(99)01558-9 PMC4126166 10375687

[CR47] Wong CT, Morgan EH (1973) Placental transfer of iron in the guinea pig. Q J Exp Physiol Cogn Med Sci 58:47–58. 10.1113/expphysiol.1973.sp002190 10.1113/expphysiol.1973.sp002190 4486760

[CR48] Xie D,Wang H, Liu Z, Fang J, Yang T, Zhou S, Wang A,Qin J, Xiong L (2017) Perinatal outcomes and congenital heart defect prognosis in 53313 non-selected perinatal infants. PloS one 12(6):e0177229. 10.1371/journal.pone.0177229 PMC5462529 28591192

[CR49] Yang H, Sun Y, Zheng X (2010) Manganese-induced apoptosis in rat myocytes. J Biochem Mol Toxicol 21:94–100. 10.1002/jbt.20172 10.1002/jbt.20172 17623882

[CR50] Yang J, Kang Y, Cheng Y, Zeng L, Dang S (2019) Iron intake and iron status during pregnancy and risk of congenital heart defects: a case-control study. 301 10.1016/j.ijcard.2019.11.115 31767385

[CR51] Yang J, Kang Y, Cheng Y, Zeng L, Shen Y, Shi G, Liu Y, Qu P, Zhang R, Yan H, Dang S (2020). Iron intake and iron status during pregnancy and risk of congenital heart defects: a case-control study. Int J Cardiol.

[CR52] Zou GY (2008). On the estimation of additive interaction by use of the four-by-two table and beyond. Am J Epidemiol.

